# 

**Published:** 1838

**Authors:** 


### WESTERN JOURNAL

OF THE

### MEDICAL AND PHYSICAL SCIENCES:

EDITED AND PUBLISHED, QUARTERLY,

BY THE

MEDICAL FACULTY

OF

THE CINCINNATI COLLEGE.

VOL. XII. NO. XLV.

SECOND HEXADE, VOL. VI. No. I. JULY, 1838,

CINCINNATI, OHIO:
PUBLISHED AT THE CINCINNATI HOSPITAL,
OPPOSITE THE COLLEGE EDIFICE, WALNUT STREET.
1838.

